# Climate-Driven Shifts in Bat Distributions Reveal Functional Reorganization and Spatial Mismatch Across Agroecosystems

**DOI:** 10.3390/biology14111528

**Published:** 2025-10-30

**Authors:** Yingying Liu, Yang Geng, Yushi Pan, Hao Zeng, Zhenglanyi Huang, Peter John Taylor, Tinglei Jiang

**Affiliations:** 1 Jilin Provincial Key Laboratory of Animal Resource Conservation and Utilization, Northeast Normal University, 2555 Jingyue Street, Changchun 130117, China; liuyy777@nenu.edu.cn (Y.L.);; 2 College of Life Science, Jilin Agricultural University, 2888 Xincheng Street, Changchun 130118, China; 3 Department of Zoology & Entomology & Afromontane Research Unit, University of the Free State, QwaQwa Campus, Private Bag X13, Phuthaditjhaba 9866, South Africa

**Keywords:** DNA metabarcoding, ecosystem services, climate change, species distribution modeling, habitat suitability

## Abstract

**Simple Summary:**

Climate change is shifting where species live, and this can disrupt important ecosystem services—like how insect-eating bats help control crop pests. We studied the Asian long-fingered bat, *Miniopterus fuliginosus*, to understand how future climate might affect its ability to protect crops. First, we used DNA analysis on bat feces from three different Chinese regions. We found that these bats mainly eat moths, including many pests that harm rice and maize, and their diet stays consistent across all areas—showing that they reliably help control pests. Then, we used models to predict the bats’ current suitable habitats and those in the 2050s and 2070s, under two climate scenarios: one with low greenhouse gas emissions (less warming) and one with high emissions (more warming). The models showed that the bats’ suitable habitats will shift north. While the total suitable area may stay stable or grow, high emissions will split habitats into small, isolated patches. This could mean that bats will not be where their pest control is most needed for crops. Our work helps identify areas to protect (where bats can survive and control pests) and guide plans for connected habitats.

**Abstract:**

Understanding how climate change may reshape species distributions and affect the associated ecosystem services is critical for sustainable agricultural planning. In this study, we integrated dietary DNA metabarcoding with ensemble species distribution modeling to assess the current and future ecological roles of *Miniopterus fuliginosus*, a widespread insectivorous bat species in East Asia known for preying on nocturnal agricultural pests. Fecal samples were collected in 2023 from three biogeographically distinct regions of China—Central China (Henan Province) and Southwest China (Guizhou and Yunnan provinces). DNA metabarcoding based on COI gene amplification and Illumina sequencing revealed a consistent dietary dominance of Lepidoptera, particularly families comprising major agricultural pest species such as Noctuidae, Crambidae, and Geometridae. This trophic consistency suggests that *M. fuliginosus* functions as a moth-specialized generalist predator. Species distribution models were constructed using occurrence records from field surveys, the literature, and the GBIF database, integrating multiple algorithms (GLM, GBM, MaxEnt, RF, and FDA) within an ensemble modeling framework. Habitat suitability was then estimated under current climatic conditions and projected for future distributions under two contrasting climate scenarios (SSP1–2.6 and SSP5–8.5) for the 2050s and 2070s. While the total suitable area may remain stable or even expand, future projections indicate a progressive poleward shift in range centroids and a divergence in habitat structure. Specifically, SSP1–2.6 is associated with greater spatial cohesion (25.34–31.11%), whereas SSP5–8.5 leads to increased habitat fragmentation and isolation of suitable patches (27.12–33.28%). Overlaying the potential for pest control with habitat projections highlights emerging spatial mismatches between ecological function and climatic suitability, particularly under high-emission trajectories. Our findings underscore the importance of identifying ecological refugia and maintaining landscape connectivity to sustain bat-mediated pest control. This spatially explicit framework offers new insights for integrating biodiversity-based pest management into climate-resilient agricultural strategies.

## 1. Introduction

Climate change is reshaping the spatial organization of global biodiversity at an unprecedented rate, altering the climatic envelopes within which species can persist [1]. As mean temperatures rise and seasonal precipitation patterns change, many taxa are undergoing directional range shifts—typically poleward or upslope—as they track suitable climatic conditions [2]. While such shifts may reduce the immediate risk of extinction, they also generate uncertainty concerning where ecological functions will continue to be delivered [3,4]. This is particularly concerning for mobile species whose functional roles, such as pest control or pollination, depend on their co-occurrence with specific habitat types or resource distributions [5].

Bats, as highly mobile vertebrates, provide diverse ecological functions but are particularly sensitive to environmental change [6]. As volant mammals, insectivorous bats in particular occupy narrow thermal niches, possess limited energy reserves, and depend on ephemeral insect prey, making them especially vulnerable to both climatic shifts and land-use disturbances [7,8]. Bats are increasingly recognized as providers of key ecosystem services—including natural pest regulation in agricultural landscapes—yet it remains unclear whether such services will persist as climate change alters species distributions and agroecological zones [9,10]. Recent evidence shows that climate change is already reshaping bat ecology by driving range shifts, altering phenology, and increasing mortality during extreme events [7,11]. Understanding how climate change may disrupt the spatial matching between bat activity and ecosystem services is essential for designing effective conservation strategies in a rapidly changing world.

Among Asia’s diverse bat fauna, the Asian long-fingered bat (*Miniopterus fuliginosus*) is one of the most widely distributed cave-roosting species, occurring across a broad latitudinal gradient from temperate to subtropical regions [12,13]. This medium-sized insectivorous bat typically roosts in humid caves and emits frequency-modulated echolocation calls adapted for open-space foraging. Although listed as a species of Least Concern by the IUCN, it faces localized threats from habitat disturbance, cave tourism, and pesticide exposure. The species is frequently observed foraging over agricultural landscapes—particularly rice and maize fields—indicating its potential role in regulating nocturnal insect populations [14]. Despite its ecological ubiquity and apparent association with pest-rich habitats, our understanding of the functional role of *M. fuliginosus* in ecosystems remains limited. In particular, little is known about the taxonomic specificity of its prey, the spatial consistency of its trophic behavior across environmental gradients, and the extent to which its ecological function may persist as climate and land-use patterns shift. Existing studies on *M. fuliginosus* have largely addressed either dietary composition or habitat suitability in isolation, without integrating these dimensions to assess the resilience of ecosystem service delivery under climate change. This disconnect hinders our ability to anticipate whether such key service-providing species will remain functionally aligned with the systems that depend on them.

Aerial insectivorous bats are increasingly acknowledged as key contributors to natural pest control, particularly in tropical and subtropical agroecosystems where chemical inputs are often intensive and ecologically disruptive. Studies from North America and Europe have demonstrated that bat predation can significantly reduce the populations of major nocturnal crop pests, including moths in the families Noctuidae and Crambidae [15,16]. These services not only enhance crop yields but also provide substantial economic value by reducing the reliance on pesticides [9,15]. Despite this growing body of evidence, the functional roles of such species remain underdocumented in Asia, especially for widely distributed, behaviorally adaptable species such as *M. fuliginosus*. This species frequently forages over rice and maize fields—crops that are particularly susceptible to damage from Lepidopteran pests such as *Spodoptera litura* and *Mythimna separata*—yet few studies have quantified its dietary composition in relation to agricultural pest assemblages. DNA metabarcoding—an application of high-throughput sequencing to environmental samples—enables the high-resolution identification of prey taxa from bat guano, offering comprehensive insights into dietary composition and trophic interactions [17]. Bridging this knowledge gap is essential to determine whether *M. fuliginosus* plays a sustained ecological role in pest control across spatial and environmental gradients and whether that role can be expected to persist under future climate scenarios.

To evaluate the spatial resilience of bat-mediated pest control under climate change, we developed an integrative framework that combines dietary DNA metabarcoding with ensemble species distribution modeling (SDM). Ensemble species distribution modeling (SDM) integrates predictions from multiple algorithms to reduce model-specific biases and improve robustness and accuracy compared to single-model approaches [18]. Fecal samples were collected from three biogeographically distinct populations of *M. fuliginosus* in China to characterize the prey taxonomic composition and diet breadth of the species, with particular emphasis on economically important Lepidopteran pests. Future climate projections were based on two contrasting Shared Socioeconomic Pathways (SSP1–2.6 and SSP5–8.5), representing low- and high-emission scenarios, respectively. SSP1–2.6 assumes strong mitigation and sustainable development leading to limited global warming (<2 °C by 2100), whereas SSP5–8.5 reflects a fossil-fuel-intensive trajectory with high greenhouse gas emissions and continued warming exceeding 4 °C [19]. To capture near- and long-term trends in climatic suitability, projections were evaluated for two standard time horizons—mid-century (2041–2060) and late century (2061–2080)—which correspond to the IPCC’s CMIP6 climate assessment intervals commonly used for species distribution modeling and biodiversity forecasting. This approach links trophic data with spatial modeling to identify not only where the species may persist but also where its ecological function is most likely to be maintained.

In this study, we distinguish between species conservation—the persistence of populations under changing climates—and functional conservation—the maintenance of ecological roles such as pest suppression. While species conservation focuses on preventing range contraction or extinction, functional conservation emphasizes sustaining the ecosystem services that species provide to agricultural landscapes. This study addresses three interrelated questions. (1) Does *M. fuliginosus* exhibit consistent trophic specialization on pest taxa across distinct environmental regions, and how stable is its ecological role in agroecosystems? (2) How will future climate scenarios affect the suitable habitat range of the species, and will these changes disrupt the spatial congruence between bat occurrence and potential pest control? (3) To what extent will shifts in habitat area, the range centroid, and landscape connectivity lead to spatial functional mismatches, and where might ecological refugia persist under future climatic conditions? By explicitly coupling dietary ecology with projected distributional change, we move beyond species persistence as a conservation endpoint and instead evaluate the stability of biodiversity-driven ecosystem services in changing agricultural landscapes.

## 2. Materials and Methods

### 2.1. Study Sites and Sample Collection

**Figure 1:**
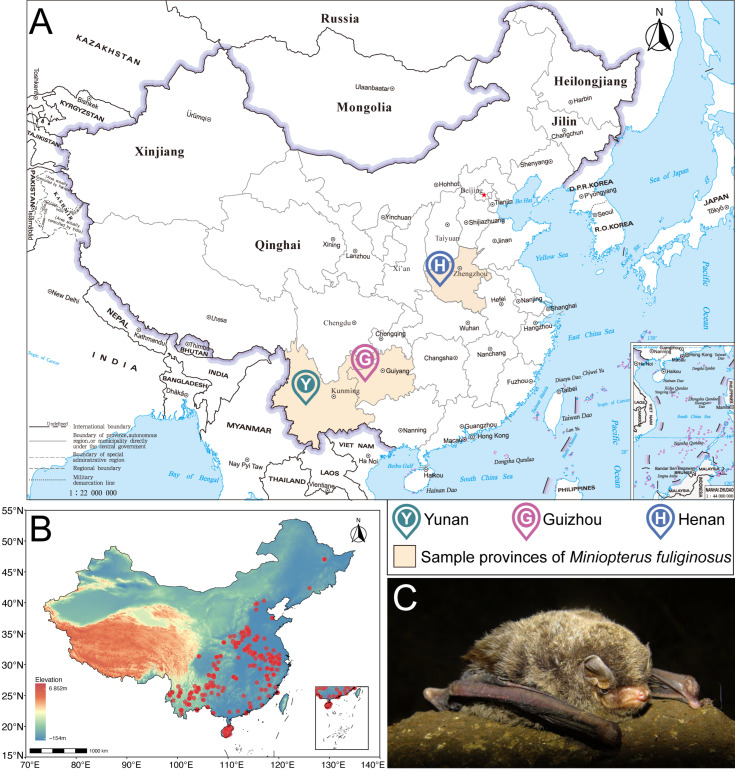
Sampling and occurrence locations of *Miniopterus fuliginosus* used in this study. (**A**) Geographic locations of fecal sampling sites for dietary analysis (Henan, Guizhou, and Yunnan provinces). (**B**) Verified species occurrence records used for species distribution modeling (SDM). (**C**) Representative photograph of *M. fuliginosus* (photographed by Congnan Sun).

Field sampling was conducted from May to August 2023, corresponding to the peak foraging period of *M. fuliginosus* and the highest seasonal abundance of nocturnal insects (Figure 1A). Three sites were selected across distinct climatic and ecological zones in China: Henan (Central China), characterized by temperate monsoon climate and mosaic cropland–forest landscapes; Guizhou (Southwest China), dominated by subtropical evergreen broadleaf forests and karst terrain; and Yunnan (Southwest China), featuring a subtropical plateau climate with mixed agricultural and forest habitats. These sites were chosen based on the long-term monitoring records of stable *M. fuliginosus* roosting colonies, their proximity to agricultural landscapes, and accessibility for field sampling. Sampling was conducted in July 2023, during the summer season, which coincides with the peak abundance of nocturnal insects and the period of the most intense foraging activity of bats. Individuals of *M. fuliginosus* were captured using mist nets placed at the entrances of known day roosts. Each site was surveyed once during this period to ensure temporal consistency, and fecal samples were collected non-invasively from captured individuals before release. Trapping was performed during the bats’ return flights at dawn, between approximately 00:30 and 02:30 local time. Immediately after capture, individuals were held temporarily in clean and sterile paper bags, and freshly defecated pellets were collected using sterile forceps. Only one fecal sample was collected per individual to avoid pseudoreplication [20]. All fecal samples were individually stored in sterile 2 mL microcentrifuge tubes, kept on ice in the field, and subsequently stored at −80 °C in the laboratory until DNA extraction. At each sampling site, we selected roosts with estimated colony sizes of at least 50 individuals to ensure representative coverage across regions and minimize local environmental bias. Across the three sampling sites, a total of 32 individual fecal samples were successfully collected and analyzed, with sample numbers varying among sites due to differences in colony size and cave accessibility. All field procedures adhered to the ethical guidelines for handling wild bats and were approved by the Institutional Animal Care and Use Committee of Northeast Normal University (Approval No. 202301001).

### 2.2. DNA Extraction and PCR Amplification

The QIAamp Fast DNA Stool Mini Kit (Qiagen, Hilden, Germany) was used to extract total DNA from individual fecal pellets according to the manufacturer’s protocol, with slight modifications to optimize DNA yield from degraded samples. Each fecal sample was homogenized before extraction, and blanks were included in every batch to monitor potential contamination [21]. To amplify arthropod DNA fragments, we used a universal primer pair targeting the cytochrome c oxidase subunit I (COI) region: LCO1490 (5′-GGTCAACAAATCATAAAGATATTGG-3′) and ZBJ-ArtR2c (5′-WACTAATCAATTWCCAAATCCTCC-3′) [22]. To monitor potential cross-contamination, two negative controls containing no DNA were included during the DNA extraction, PCR amplification, and library preparation steps, following the procedures described by Alberdi et al. [23]. Paired-end sequencing (2 × 300 bp) was performed on an Illumina MiSeq platform (Illumina Inc., San Diego, CA, USA) at Majorbio Bio-Pharm Technology Co., Ltd. (Shanghai, China).

### 2.3. Bioinformatic Analysis and Taxonomic Assignment

The raw sequence data were demultiplexed and processed using QIIME2 (version 2022.8) and OBITools3 [24,25]. The paired-end reads were merged, quality-filtered (Q score > 30), dereplicated, and clustered into operational taxonomic units (OTUs) at a 97% similarity threshold using USEARCH [26]. The uchime_denovo algorithm was used to remove chimeric sequences [27]. Representative sequences of each OTU were taxonomically assigned using BLASTn v2.13.0 (National Center for Biotechnology Information, Bethesda, MD, USA) searches against a custom reference database compiled from BOLD and GenBank, with priority given to high-confidence arthropod entries from East Asia [28]. Sequences were identified to the species level at ≥98% identity, to the genus level at ≥97%, and to the family level at ≥95% [29]. We cross-referenced identified taxa with established Chinese agricultural pest species lists to annotate which prey species were known crop pests [30,31,32,33,34].

To assess spatial variation in the dietary composition and trophic niche structure of *M. fuliginosus*, we performed a series of analyses based on relative read abundance data using R (v4.3.0). Non-metric multidimensional scaling (NMDS) based on Bray–Curtis dissimilarity was used to visualize inter-regional differences, with significance tested via ANOSIM (999 permutations) [14]. We calculated the Shannon diversity index and Levins’ standardized niche breadth (Bsta) to quantify within-population diversity and Pianka’s niche overlap index to assess dietary similarity between populations [35]. Additionally, β-diversity was partitioned into turnover and nestedness components using the betapart package (1.6.1) for R to identify the driving factors of inter-population differences in diet [36].

### 2.4. Species Occurrence Data and Environmental Predictors

To model the current and future distribution of *M. fuliginosus*, we compiled a comprehensive database of georeferenced occurrence records from three primary sources. First, we incorporated our field survey data, collected between 1995 and 2024, across the majority of Chinese provinces, with particular emphasis on limestone karst landscapes and other known or potential roosting habitats. Second, we extracted published occurrence records from the peer-reviewed literature and regional faunal inventories. Third, we downloaded publicly available records from the Global Biodiversity Information Facility (GBIF) [37]. All records were manually screened to eliminate duplicates and spatial errors. Only records with a coordinate uncertainty of less than 1 km were retained to ensure spatial accuracy and comparability across data sources. To reduce spatial sampling bias and account for potential autocorrelation in highly clustered areas, we implemented a spatial thinning procedure using the spThin package (0.2.0) in R [38]. A 10 km thinning distance was applied, allowing for only a single presence record within each 10 km radius [39]. This approach is commonly used to reduce pseudoreplication in species distribution modeling. To reduce spatial autocorrelation among occurrence records, we applied a spatial thinning distance of 10 km, retaining only one presence point within each 10 km radius. This distance was selected based on high-precision GPS tracking data from our ongoing research and previous studies on *M. fuliginosus*, which indicate that this species typically forages within an 8–12 km radius around its day roosts. Thus, a 10 km thinning radius provides an ecologically meaningful scale that approximates the species’ average home range while ensuring spatial independence among occurrence points. A total of 699 occurrence records were compiled before spatial thinning, and 312 spatially independent records were retained after applying a 10 km thinning distance (Supplementary Table S1).

A set of environmental predictors was selected based on ecological relevance to bat physiology, roosting behavior, foraging habitat preference, and potential responses to climate and land-use changes. Specifically, we included 19 bioclimatic variables (BIO1–BIO19) from WorldClim v2.1 at a 30 arc-second (~1 km) spatial resolution [40]. These variables described annual trends, seasonality, and extreme conditions in temperature and precipitation. To account for land-use and habitat heterogeneity, we extracted land cover data from the MODIS MCD12Q1 product, including classes such as cropland, forest, grassland, and urban areas [41]. Additionally, we included the Global Human Footprint Index (version 2, 2009) as a proxy for anthropogenic pressure [42], as well as topographic layers (elevation and slope) derived from the Shuttle Radar Topography Mission Digital Elevation Model at 1 km resolution.

All environmental rasters were resampled to a common resolution of 1 km using bilinear interpolation, and the resulting distributions were spatially clipped to the terrestrial boundary of mainland China. To reduce multicollinearity among predictors, we calculated variance inflation factors (VIFs) using the usdm package in R [43,44]. NDVI data were extracted from the MODIS MOD13A3 dataset (Terra, NASA; Version 6; 1 km resolution; 2000–2023) and scaled by 1 × 10^−4^. Four NDVI-derived variables were calculated to represent vegetation productivity and temporal variability: annual maximum NDVI (*NDVImax*, indicating vegetation growth potential), annual mean NDVI (*NDVImean*, representing average productivity), inter-annual standard deviation (*NDVIysd*, reflecting year-to-year variation), and monthly standard deviation (*NDVImsd*, representing seasonal dynamics). All NDVI layers were resampled and spatially aligned with other environmental predictors. Variables with VIF values greater than 10 were iteratively excluded from the predictor set until all remaining variables satisfied the collinearity threshold [45]. The retained NDVI metrics showed low intercorrelation (|r| < 0.7), capturing distinct aspects of vegetation dynamics relevant to habitat quality for *M. fuliginosus*. The VIF analysis retained the following environmental variables for modeling: BIO2, BIO3, BIO8, BIO15, BIO18, BIO19, karst, cropland, forest, grassland, urban, water, light, NDVImean, NDVIysd, NDVImsd, HFmean, and HFsd (see Supplementary Table S2). These predictor variables captured the key environmental gradients likely to shape the distribution of *M. fuliginosus*. All spatial data processing, raster manipulation, and variable extractions were conducted using ArcGIS 10.7 and R version 4.3.0, utilizing the raster and terra packages for geospatial analyses [46,47,48].

### 2.5. Species Distribution Modeling and Projections

We used the R package biomod2 (4.2-6-2) [49] to develop ensemble SDMs for *M. fuliginosus* (Figure 1B). Five algorithms were used to capture methodological variation: generalized linear models (GLMs), generalized boosted models (GBMs), maximum entropy (MaxEnt), random forest (RF), and flexible discriminant analysis (FDA). Occurrence records were filtered to minimize spatial autocorrelation and then randomly partitioned into 80% training and 20% testing datasets [50,51]. Five-fold cross-validation was used to evaluate model performance. For each algorithm, only models with a receiver operating characteristic (ROC) score > 0.85 were retained [52]. A weighted mean approach was used to generate ensemble predictions, with model weights based on their True Skill Statistic (TSS) performance [53]. The final ensemble outputs were continuous habitat suitability maps, with values ranging from 0 to 1.

To classify and compare the spatial patterns of suitability, the ensemble maps were reclassified into four habitat categories using the natural breaks (Jenks) method: unsuitable (0–0.1), marginally unsuitable (0.1–0.29), marginally suitable (0.29–0.5), and highly suitable (>0.5). This classification was applied to both current and future predictions [54]. The centroids of suitable habitats (across different habitat categories) under current and future scenarios were calculated using the xyFromCell function in the terra package, and migration distance was computed by using the raster package [46,47,48]. All spatial analyses and reclassifications were conducted in R using the raster and classInt packages (0.4–11). The final set of predictors, selected via VIF analysis, included both bioclimatic and anthropogenic variables. To assess the potential impacts of climate change, the ensemble models were projected onto future bioclimatic layers derived from the CMIP6 dataset under two Shared Socioeconomic Pathways (SSP1–2.6 and SSP5–8.5), for two time periods: mid-century (2041–2060) and late century (2061–2080) [55,56]. To ensure temporal consistency, only the bioclimatic variables from WorldClim v2.1 were used to project current and future habitat suitability under both SSP scenarios. Anthropogenic and landscape variables (e.g., land-use, NDVI, and human footprint) were incorporated separately in post-model correlation analyses to interpret present-day environmental associations but were not included in future projections, as reliable global layers for these predictors are not available for the SSP framework. All environmental variables were standardized to a 1 km spatial resolution and cropped to the terrestrial boundaries of mainland China.

## 3. Results

### 3.1. Dietary Composition Reveals Strong Specialization on Lepidoptera and Agricultural Pests

**Figure 2:**
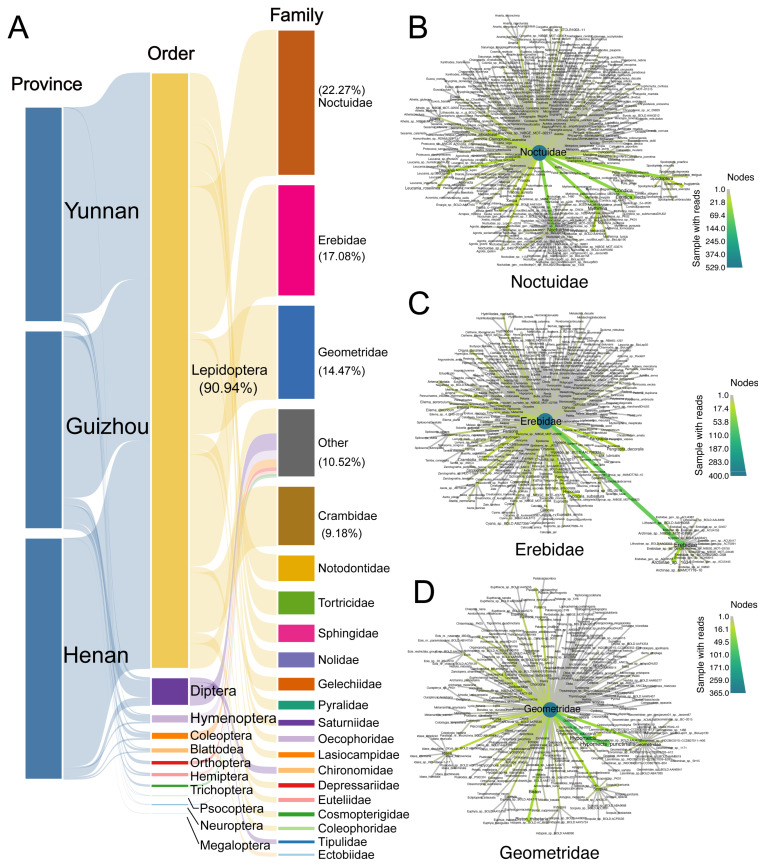
Dietary composition and taxonomic network structure of *Miniopterus fuliginosus* based on DNA metabarcoding of guano samples from three provinces in China. (**A**) Sankey diagram showing proportional representation of prey taxa from province to order and family levels. Lepidoptera dominated diet (90.94% of total reads), primarily composed of Noctuidae (22.27%), Erebidae (17.08%), Geometridae (14.47%), Crambidae (9.18%), and other minor families. (**B**–**D**) Taxonomic co-occurrence networks of dominant Lepidopteran families—(**B**) Noctuidae, (**C**) Erebidae, and (**D**) Geometridae. Each node represents prey species, with node size indicating number of samples in which taxon was detected. Edge thickness reflects co-occurrence frequency among taxa, and color gradients denote number of samples containing each species.

The dietary profiling of *M. fuliginosus* (Figure 1C) based on the DNA metabarcoding of fecal samples from 32 individuals revealed a highly uneven prey composition dominated by Lepidoptera. At the order level, Lepidoptera accounted for 90.94% of the total sequencing reads across all samples (Figure 2A), primarily comprising families Noctuidae (22.27%), Erebidae (17.08%), Geometridae (14.47%), Crambidae (9.18%), and Notodontidae, indicating a strong and consistent trophic bias toward Lepidopteran prey. This pattern was stable across all three sampled regions (Henan, Guizhou, and Yunnan), with only minor contributions from Diptera (4.43%), Coleoptera (1.66%), Hemiptera (1.21%), and other arthropod groups, each contributing less than 1% of the total reads. The consistently low representation of non-Lepidopteran taxa underscores the pronounced selectivity of *M. fuliginosus* for Lepidoptera.

At the family level (Figure 2B), Noctuidae was by far the dominant group, comprising 69.4% of the total reads, followed by Erebidae, Geometridae, and Crambidae. These families include many well-known agricultural pests with high nocturnal activity and strong attraction to light, making them particularly vulnerable to aerial insectivores. The predominance of Noctuidae was observed in nearly all individuals, with many samples containing multiple noctuid genera and species. Genus-level resolution revealed that *Spodoptera*, *Mythimna*, *Cnaphalocrocis*, *Helicoverpa*, and *Agrotis* were among the most frequently detected and abundant genera in the dataset (Figure 2C). These genera include key pest species such as *Spodoptera litura*, *Mythimna separata*, and *Cnaphalocrocis medinalis*, species that are notorious for their economic impacts on rice, maize, and vegetable cultivation throughout East Asia. Notably, these species were detected not only at high read abundance but also in a wide range of individuals and across all three regions, suggesting their role as consistent core prey taxa. A species-level analysis of the noctuid component (Figure 2D) further confirmed that *M. fuliginosus* frequently consumed a diverse array of pest species, including *S. litura*, *M. separata*, *H. armigera*, *A. ipsilon*, and *C. medinalis*, all of which are major targets of chemical pest control in agroecosystems. The broad representation of these pests in the bats’ diet provides direct evidence of their functional role in suppressing nocturnal insect pests.

In sum, the dominance of Lepidoptera across taxonomic levels, the consistently high representation of pest families and genera, and the wide spatial coverage of these patterns collectively support the conclusion that *M. fuliginosus* functions as a moth-specialized generalist predator. This foraging strategy combines a broad dietary spectrum within Lepidoptera with consistent prey-type composition across geographically distinct agricultural populations, suggesting that the species occupies a specialized dietary niche within subtropical agroecosystems and contributes to pest control.

### 3.2. Regional Differences in Dietary Composition and Niche Metrics

**Figure 3:**
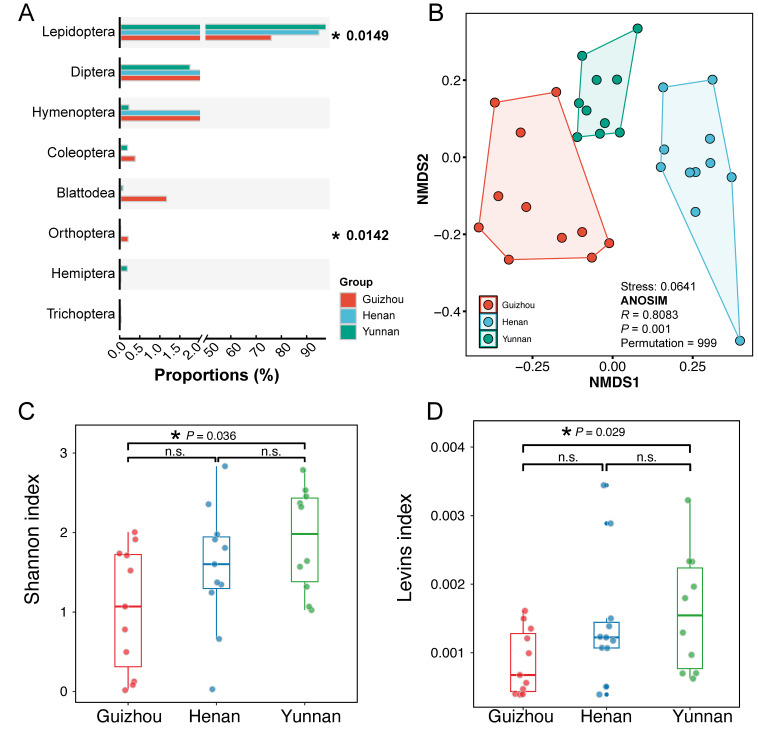
Regional variation in the dietary composition of *Miniopterus fuliginosus***.** (**A**) The relative read abundance (%) of the six most frequently detected arthropod orders across three provinces (Guizhou, Henan, and Yunnan). Bars represent group means. Asterisks indicate significant overall differences among regions based on Kruskal–Wallis tests (*p* < 0.05). (**B**) A non-metric multidimensional scaling (NMDS) plot based on the Bray–Curtis dissimilarity of prey composition. (**C**) Box plots of prey diversity (Shannon index). (**D**) Box plots of dietary niche breadth (Levins index). Each point represents an individual fecal sample. ‘n.s.’ indicates non-significant differences (*p* > 0.05).

The dietary composition of *M. fuliginosus* exhibited significant geographic differentiation across the three sampled regions. Non-metric multidimensional scaling (NMDS) ordination based on Bray–Curtis dissimilarity demonstrated clear separation among populations from Henan, Guizhou, and Yunnan (Figure 3A). The ANOSIM confirmed significant among-group divergence (R = 0.808; *p* = 0.001), indicating strong regional structuring in prey assemblages. Despite the predominance of Lepidoptera in all regions, the proportional representation of other prey orders varied markedly (Figure 3B). Guizhou individuals showed higher relative abundances of Diptera and Mesostigmata, while Yunnan bats consumed a broader range of arthropods, including Trichoptera and Orthoptera. Araneae and Blattodea were detected at low frequencies in all populations.

Diversity metrics revealed significant inter-regional differences in trophic heterogeneity. The Shannon diversity indices differed across provinces (*p* = 0.036), with Yunnan bats exhibiting the highest within-population prey diversity (Figure 3C). The standardized Levins’ niche breadth index also varied significantly (*p* = 0.029), with the highest values in Yunnan and the lowest in Henan (Figure 3D), suggesting broader dietary niche space in the southern population. Collectively, these results indicate that *M. fuliginosus* exhibits spatially structured foraging patterns, with region-specific variation in both prey taxonomic composition and dietary niche width.

### 3.3. Model Evaluation, Variable Importance, and Current Habitat Suitability of Miniopterus fuliginosus

**Figure 4:**
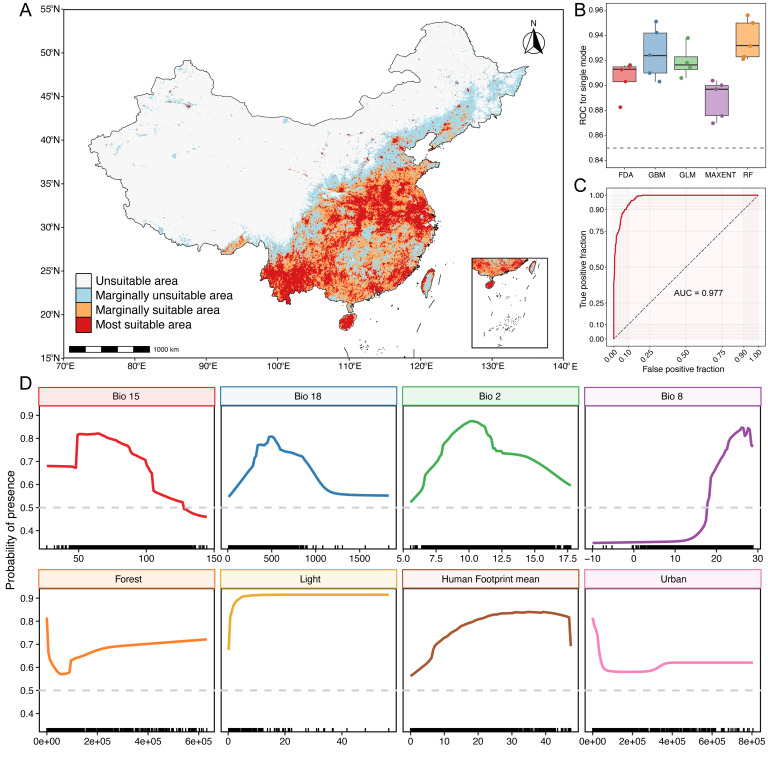
Current and projected distributions and model performance for *Miniopterus fuliginosus*. (**A**) Current predicted distribution of suitable habitat based on ensemble species distribution models. (**B**) ROC scores for five algorithms used in model construction. (**C**) AUC values for ensemble species distribution model. (**D**) Response curves of top eight environmental predictors derived from ensemble species distribution model. Each panel illustrates marginal effect of single predictor variable on probability of presence while holding other variables constant.

The ensemble SDM constructed for *M. fuliginosus* achieved high predictive accuracy across all evaluation metrics (Figure 4). The ensemble model yielded a mean area under the receiver operating characteristic curve (AUC) of 0.977 and a mean TSS of 0.839, indicating excellent discriminatory capacity between presence and background points (Figure 4A). All five individual algorithms—GLM, GBM, MaxEnt, RF, and FDA—produced robust results, with AUC scores ranging from 0.870 to 0.956 (Figure 4B). These performance scores confirmed the reliability and consistency of the ensemble model in capturing the environmental constraints of the species distribution.

An analysis of variable importance revealed that both climatic and land-use factors contributed significantly to predicting habitat suitability (Figure 4C). Among the bioclimatic predictors, precipitation seasonality (BIO15) was the most influential, followed by the precipitation of the warmest quarter (BIO18), the mean temperature of the wettest quarter (BIO8), and temperature seasonality (BIO2). These variables characterize seasonal water availability and thermal stability, key climatic dimensions that likely constrain the availability of insect prey and the microclimates of roost sites. Among anthropogenic and landscape-level variables, forest cover and the proportion of cropland positively influenced habitat suitability. Night-time light intensity showed a monotonic, saturating increase in suitability, whereas the human footprint exhibited a unimodal relationship, with habitat suitability increasing under moderate human influence and declining at the highest disturbance levels, indicating that *M. fuliginosus* avoids highly urbanized and disturbed areas (Figure 4D). Notably, the presence of karst terrain and vegetation productivity metrics (e.g., NDVImean and NDVIysd) also showed moderate importance, consistent with the species’ known reliance on cave roosts and foraging in heterogeneous, semi-natural landscapes.

The final ensemble projection of current habitat suitability identified extensive areas of high suitability (suitability score > 0.5) across southern and southeastern China, encompassing approximately 1.70 × 10^6^ km^2^. Core regions include Guangxi, Guangdong, Fujian, Hunan, Jiangxi, Guizhou, and Yunnan (Figure 4A). These areas form a contiguous band of climatically favorable and structurally heterogeneous habitats characterized by warm, humid conditions; extensive karst systems; and agroforestry mosaics. Moderately suitable areas (suitability 0.29–0.5) were distributed in a transitional zone extending northward into southern Henan, Anhui, Chongqing, and eastern Sichuan. Under future climate scenarios, the total suitable area is projected to expand to 2.10 × 10^6^ km^2^ by the 2050s and then slightly contract to 1.79 × 10^6^ km^2^ by the 2070s under SSP1–2.6, indicating an initial gain followed by stabilization. In contrast, under SSP5–8.5, suitable habitats are expected to reach 1.92 × 10^6^ km^2^ in the 2050s and 1.98 × 10^6^ km^2^ by the 2070s, reflecting a steady northward expansion into new climatic zones. In contrast, large portions of northern, northwestern, and northeastern China, including Inner Mongolia, Gansu, Qinghai, and the Northeast Plain, are predicted to be climatically unsuitable (suitability < 0.1), primarily due to cold or arid conditions and the absence of appropriate roosting habitats. Collectively, these results provide a robust baseline of the current potential distribution of *M. fuliginosus* and clarify the relative roles of climatic stability, land cover and structure, and anthropogenic disturbance in shaping its range across heterogeneous landscapes in China.

### 3.4. Climate-Driven Redistribution and Fragmentation of Suitable Habitat Across Future Scenarios

**Figure 5:**
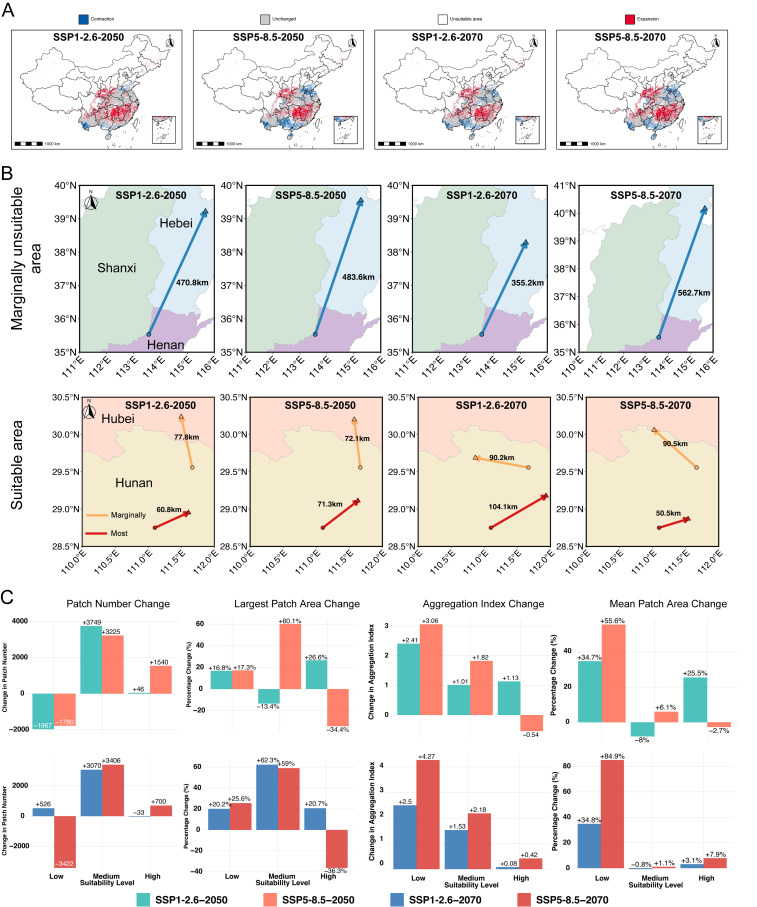
Projected changes in spatial distribution and landscape configuration of climatically suitable habitats for *Miniopterus fuliginosus* under two emission scenarios (SSP1–2.6 and SSP5–8.5) for 2050s and 2070s. (**A**) Contraction (blue), expansion (red), unchanged (gray), and unsuitable (white) areas of habitat suitability are mapped. (**B**) Latitudinal centroid shifts are shown, indicating progressive northward displacement of suitable habitat, with stronger displacement under SSP5–8.5 than under SSP1–2.6. Top row shows shifts in marginally unsuitable areas (defined as regions with habitat suitability index of 0.1–0.29), and bottom row shows shifts in suitable areas (marginally suitable areas with index of 0.29–0.5 and most suitable areas with index of 0.5–1). Arrows indicate direction and magnitude of centroid movement: blue arrows represent shifts in marginally unsuitable areas, orange arrows represent shifts in marginally suitable areas, and red arrows represent shifts in most suitable areas. Different provinces are represented with different colors. (**C**) Quantification of landscape-level changes in patch number under different scenarios, largest patch area, aggregation index, and mean patch area across low, medium, and high suitability classes. Colors distinguish scenarios: green (SSP1–2.6 2050s), orange (SSP5–8.5 2050s), blue (SSP1–2.6 2070s), and red (SSP 5–8.5 2070s).

The ensemble SDMs projected notable shifts in the distribution and structural integrity of climatically suitable habitats for *M. fuliginosus* under both low-emission (SSP1–2.6) and high-emission (SSP5–8.5) scenarios across mid- (2050s) and late-century (2070s) timeframes. The most consistent pattern across all scenarios was a poleward redistribution of suitable habitat, with expansion into the North China Plain and the southern Loess Plateau and concurrent contractions across the humid subtropical lowlands of southern China (Figure 5A).

Spatial changes were moderate and relatively stable under the SSP1–2.6 scenario. By the 2050s, newly suitable areas will emerge primarily in southern Henan, northern Anhui, and central Jiangsu, while habitat loss will concentrate in eastern Yunnan and Guizhou. These trends will intensify slightly by the 2070s, with expansion continuing into southern Shanxi and northern Hubei. Under the SSP1–2.6 scenario, habitat dynamics showed a clear temporal pattern. By the 2050s, substantial gains in suitable areas (31.1% of current extent) will far exceed losses (7.6%), resulting in a significant 23.5% net expansion (Table S3). Notably, much of the current core distribution in Central China remained climatically stable across both time segments, indicating the persistence of functional refugia. By the 2070s, the centroid of highly suitable habitats will shift northeastward by 195.6 km (Table S4), reinforcing the poleward trend. However, by the 2070s, this trend will be moderated considerably, with gains (25.34%) only slightly exceeding losses (19.5%), yielding a modest 5.8% net increase. This temporal pattern suggests that while initial climate change may create new suitable habitats, continued warming leads to more balanced gains and losses (Table S5).

In contrast, the projections for SSP5–8.5 revealed a stronger and more spatially extensive redistribution. By the 2050s, gains in suitable areas (27.13%) will exceed losses (13.91%), and large-scale contraction will occur across southern Hunan, Jiangxi, and western Guangxi, while expansion will occur in eastern Henan, Shandong, and southern Hebei. These trends will intensify by the 2070s, and gains in suitable areas (33.28%) will exceed losses (16.77%), with continued gains in central Shanxi and losses in the southern range edge. Although the total area of highly suitable habitats increased by 16.4% (Table S3), the expansion occurred predominantly in climatically novel northern regions, resulting in a centroid shift of 316.4 km, more than 60% greater than that under SSP1–2.6 (Table S4). However, this spatial gain was accompanied by structural degradation: the number of suitable habitat patches increased by 62.3%, while the mean patch size, the largest patch area, and the aggregation index all declined (Table S5), indicating heightened habitat fragmentation. These results were corroborated by the longitudinal trend in landscape structure (Figure S1), which shows increasing patchiness under the high-emission trajectory.

Centroid displacement analysis further revealed divergent dynamics across the scenarios and suitability classes (Figure 5B). For marginally suitable areas, all four scenarios exhibited pronounced northward displacement, ranging from 355.2 km to 562.7 km, with the greatest shift projected under SSP5–8.5 in the 2070s. In contrast, highly suitable core areas exhibited smaller but directionally variable centroid shifts (50.5–104.1 km), suggesting greater structural inertia but also higher vulnerability to fragmentation under high-emission scenarios. Taken together, these results indicate that while *M. fuliginosus* may experience net habitat expansion under future climate change, the spatial coherence and functional connectivity of these habitats diverge sharply between scenarios. SSP1–2.6 supports the maintenance of spatially cohesive ecological networks and the persistence of existing service zones. In contrast, SSP5–8.5 promotes range expansion into disaggregated northern landscapes, heightening the risk of spatial decoupling between the presence of the species and its ecological role in pest control.

### 3.5. Structural Responses of Habitat Configuration Under Future Climate Scenarios

To further quantify the landscape-level impacts of climate change on habitat structure, we compared key configuration metrics across suitability levels (low, medium, and high) under the SSP1–2.6 and SSP5–8.5 scenarios for the 2070s (Figure 5C). The results revealed marked differences in fragmentation, connectivity, and patch characteristics depending on the emission trajectory and habitat quality class.

For patch number, SSP5–8.5 projected sharp increases in medium-suitability areas (+3406 patches), exceeding that of SSP1–2.6 (+3070), suggesting more extensive fragmentation under high-emission conditions. In contrast, the number of high-suitability patches decreased slightly under SSP1–2.6 (−33), while there was a modest increase under SSP5–8.5 (+700), indicating the potential isolation of high-quality zones in the latter scenario. Notably, low-suitability patches exhibited a divergent trend: a net gain under SSP1–2.6 (+526) but a pronounced loss under SSP5–8.5 (−3422), suggesting potential local extinction or homogenization in marginal zones. Changes in the largest patch area highlighted further contrast. Medium-suitability patches experienced the most substantial increases under both scenarios, with gains of +62.3% (SSP1–2.6) and +59% (SSP5–8.5), indicating the formation of large, contiguous patches with intermediate suitability. For high-suitability areas, SSP1–2.6 yielded a 20.7% increase, while SSP5–8.5 resulted in a 36.3% decline, implying the severe fragmentation of optimal habitat under a warming trajectory. The trends in the aggregation index echoed this pattern. In low- and medium-suitability zones, SSP5–8.5 promoted greater aggregation (+4.27 and +2.18, respectively), whereas high-suitability areas showed minimal change (+0.42 under SSP5–8.5 and +0.08 under SSP1–2.6), suggesting limited improvement in the spatial cohesion of critical zones. Finally, the mean patch area increased in all categories under SSP5–8.5, most strikingly in low-suitability zones (+84.9%), while high-suitability areas showed only modest gains (+7.9%), highlighting scale- and quality-specific divergence in landscape response.

Collectively, these results indicate that under high-emission scenarios, future habitat networks for *M. fuliginosus* may become more extensive but structurally fragmented, particularly in high-suitability zones that are crucial for the ecological function of the species. Conversely, low-emission trajectories support greater habitat cohesion and continuity, especially for core habitats, emphasizing the importance of climate mitigation for functional landscape integrity.

## 4. Discussion

Our study combined dietary metabarcoding and species distribution modeling to assess the status of the ecological function and climatic vulnerability of *M. fuliginosus* in China. We demonstrated that the species exhibited a consistent trophic bias toward Lepidoptera, with economically important species of moths dominating its diet across various regions. Despite local variation in prey diversity, this functional specialization was spatially stable. Current habitat suitability is concentrated in southern and southeastern China, where high dietary diversity and the potential for pest control co-occur. Under future climate scenarios, suitable habitats are projected to expand northward but with divergent structural outcomes: SSP1–2.6 favors moderate expansion and improved habitat cohesion, while SSP5–8.5 would result in greater range displacement, increased habitat fragmentation, and loss of habitat connectivity. These results suggest that climate change may not only shift the distribution of *M. fuliginosus* but also disrupt the spatial alignment between its persistence and the delivery of ecosystem services.

### 4.1. Functional Trophic Specialization: Ecological and Evolutionary Underpinnings

The consistently high representation of Lepidoptera in the diet of *M. fuliginosus* across geographically distinct populations suggests a strong functional specialization toward moth predation, despite the ability of the species to exploit a broad taxonomic prey base. Such patterns align with the concept of a “functionally biased generalist” [57], in which prey diversity is broad but filtered through stable morphological and sensory constraints. The predominance of moths in the diet is likely driven by multiple interacting traits. *M. fuliginosus* emits short-duration, frequency-modulated echolocation calls that are well suited for detecting soft-bodied, fluttering prey in open and edge-space environments [14,58]. These call structures optimize the detection of Lepidopteran wingbeat patterns and may also reduce detectability by moths equipped with tympanal defenses [59]. In addition, the agile, high-speed flight of the species enables the efficient capture of erratic-flying noctuids, further reinforcing its ecological alignment with this prey group.

Importantly, our findings align with similar trophic patterns observed in *M. schreibersii* and *Myotis myotis* across Europe and the Mediterranean Basin [60,61], where moths consistently dominate the diet under natural conditions of prey availability. Such cross-regional convergence in prey use suggests that moth specialization may be a recurrent foraging strategy among mid-sized, aerially foraging insectivorous bats. From an evolutionary perspective, the persistence of moth-biased diets across divergent environments likely reflects selective advantages associated with foraging efficiency, prey profitability, and predator–prey acoustic matching. At the community level, this form of functional filtering may also reduce dietary overlap with sympatric bat species targeting non-Lepidopteran prey [14], thereby facilitating niche partitioning and local coexistence. Together, these findings suggest that *M. fuliginosus* occupies a functionally specialized trophic niche, characterized by morphological and acoustic traits that favor moth exploitation and stabilized by evolutionary convergence in prey selection strategies across different biogeographic contexts.

### 4.2. Climate-Driven Range Shifts and Spatial Decoupling of Ecological Function

The projected distributional dynamics under future climate scenarios revealed both a northward shift in the suitable habitat of *M. fuliginosus* and an increasing risk of spatial decoupling between the persistence of the species and its ecological function. While both the SSP1–2.6 and SSP5–8.5 scenarios forecast net expansions in suitable habitat, the SSP5–8.5 projection is characterized by a significant latitudinal centroid shift and fragmentation of high-quality habitat patches. This range reorganization displaces the core distribution of the species away from southern China, where our dietary analysis showed the highest Lepidopteran diversity and relevance to pest control.

The observed divergence between climatically suitable habitats and regions of high ecological function exemplifies a growing concern in global change ecology—spatial decoupling between functional and scenopoetic niches [3]. While species distribution models indicate where a species can persist, they do not necessarily reflect where it performs key ecological roles. In our case, *M. fuliginosus* may expand into newly suitable northern landscapes, yet these areas may not sustain the same abundance or diversity of moths that underpin its pest control function. Similar mismatches have been reported in other trophic systems under climate change [62,63] and are increasingly recognized as drivers of functional instability in both temperate and tropical ecosystems. Nevertheless, this interpretation assumes that prey distributions remain relatively stable; if moth communities also shift with changing climate, the extent and spatial pattern of this decoupling may be altered or reduced.

In the case of *M. fuliginosus*, the velocity and extent of projected range shifts may exceed the dispersal capacity of the species and the continuity of roosting habitats, particularly in fragmented agricultural landscapes [64,65]. Such constraints could hinder the species from reaching newly suitable zones in time to maintain the current functional alignment. Even if dispersal is successful, the loss of ecological matching between predator presence and pest outbreak zones could reduce the realized pest control in both current and future agricultural regions. These findings underscore the need to move beyond species persistence metrics in climate adaptation planning and instead incorporate the maintenance of pest suppression capacity as a conservation target [66]. In the context of *M. fuliginosus*, this requires not only identifying climatically stable habitats but also preserving areas where ecological function and climatic suitability co-occur—the so-called “functional refugia” [67]. Recognizing and managing these zones will be essential for sustaining bat-mediated pest regulation in the face of accelerating climate change.

### 4.3. Habitat Configuration, Connectivity, and Functional Integrity Under Climate Change

Although both climate scenarios projected an increase in the overall area of suitable habitat for *M. fuliginosus*, the structural quality and spatial organization of this habitat diverged sharply between pathways. Under SSP1–2.6, a modest expansion (+4.6%) was accompanied by increased landscape cohesion, reflected in a reduction in the number of habitat patches (from 21 to 17), an enlargement of the largest contiguous patch (+8.2%), and improved connectivity metrics. This pattern suggests that low-emission trajectories may allow *M. fuliginosus* to retain its ecological functionality within a more consolidated range, reducing the costs of dispersal and supporting demographic stability [68].

In contrast, the SSP5–8.5 scenario, while projecting a larger overall gain in suitable area (+16.4%), produced a more fragmented and structurally unstable landscape. The number of habitat patches increased to 25, the size of the largest patch declined (−10.9%), and the mean nearest-patch distance rose by 16%, indicating increased isolation among suitable areas. These changes occurred alongside a substantial centroid displacement (~316 km), suggesting that future high-suitability zones may emerge in novel areas disconnected from current roost networks and prey-rich habitats. This decoupling of spatial expansion from structural integrity highlights a fundamental limitation of range-based metrics: gains in total area do not necessarily translate into gains in functional habitat [69].

The fragmentation of suitable habitat not only increases extinction risk through edge effects and population isolation [70] but also undermines the ability of mobile species to track shifting climate envelopes. For bats, dispersal across fragmented landscapes may be constrained by the loss of suitable day-roosting caves and foraging corridors, particularly in regions where rapid land-use change coincides with climate-driven shifts in distribution [71,72]. These limitations may prevent *M. fuliginosus* from fully occupying newly suitable areas, despite their climatic favorability, and could result in the functional underfilling of the potential range [73]. Conservation strategies should therefore prioritize spatial cohesion and connectivity, not just total area, to safeguard ecological function under climate change. Identifying and protecting climate-resilient habitat networks that maintain core roosts and foraging grounds, particularly in regions bridging current and projected distributions, will be critical for enabling natural range tracking and minimizing functional disruption [74]. This will require integrative approaches that combine climatic suitability with structural metrics to define “functional landscapes” that remain viable under future conditions. Our results show that climate change not only alters the extent of suitable habitats but also reshapes their spatial structure. Under mild warming (SSP1–2.6), *M. fuliginosus* may maintain cohesive and functional habitat networks, whereas severe warming (SSP5–8.5) leads to pronounced fragmentation that could impair foraging efficiency and reproductive success.

Notably, the largest patch of high-quality habitat contracted by over one-third under SSP5–8.5, and the mean patch size declined despite apparent gains in total area. This structural decoupling—expansion in marginal zones paired with the fragmentation of the core habitat—could compromise ecological service delivery even if the range of species is expanded. Such findings align with emerging evidence that habitat integrity, rather than area alone, is a key determinant of functional resilience in mobile species [67]. Furthermore, the divergent trends in low- and medium-suitability zones underscore the complexity of climate-driven responses. The rapid increases in patch numbers and aggregation in marginal zones may reflect the emergence of sink habitats or transitional areas that are less suitable for sustained foraging. Without effective conservation planning, these zones may act as ecological traps, attracting bats to suboptimal areas with poor prey availability or a high level of anthropogenic disturbance [9,71].

Overall, our findings emphasize that climate-induced alterations in habitat configuration may erode the spatial integrity of bat-mediated pest control. Conservation strategies should therefore move beyond area-based targets to prioritize landscape cohesion and connectivity. Protecting large contiguous patches, maintaining cross-regional corridors, and identifying climate-resilient habitat networks that bridge current and projected distributions will be critical for enabling natural range tracking and sustaining the ecological functions of aerial insectivores under future climate change. Integrating climatic suitability with structural metrics to delineate such “functional landscapes” offers a robust framework for conserving ecological functionality under warming scenarios.

### 4.4. Toward Sustainable Bat-Mediated Ecosystem Services Under Climate Change

Our findings underscore the significance of *M. fuliginosus* as a key predator of nocturnal agricultural pests in China. However, the projected spatial mismatches between ecological service delivery and future climatic suitability raise urgent concerns for conservation planning. Sustaining bat-mediated pest control in a changing climate will require dynamic, function-focused strategies that integrate ecological roles with spatial viability. These findings underscore the need to move beyond species conservation metrics and explicitly integrate functional conservation targets—ensuring not only species survival but also the maintenance of pest suppression functions under future climates.

First, we advocate for the identification and protection of functional refugia—areas where pest control services and habitat suitability overlap, both currently and in the future [66,67]. These areas, primarily located in southern China’s agroforestry and karst landscapes, should serve as the anchor for conservation efforts through the long-term protection of roosts and prey-rich foraging zones. Second, *M. fuliginosus* should be included in Integrated Pest Management programs, especially in rice- and maize-growing regions where it targets high-impact Lepidopteran pests [9,10]. Ecologically informed agricultural policies, including support for insectivore-friendly farming practices such as preserving edge habitats and reducing light pollution, can enhance the effectiveness and resilience of bat-mediated pest control measures [71]. Third, given the consistent northeastward shift in projected habitat, maintaining landscape-scale connectivity will be crucial for the species. Targeted corridor design and the restoration of stepping-stone roosts along predicted expansion zones can facilitate range tracking and preserve functional continuity [74,75].

Although pest abundance could theoretically influence bat foraging opportunities, it was not included as a predictor in our SDM because *M. fuliginosus* exhibits high dietary plasticity and its prey composition varies with local insect availability rather than any fixed pest gradient. Moreover, consistent, spatially explicit datasets of pest abundance are lacking at the national scale. Instead, we inferred the relationship between bat distribution and pest occurrence indirectly by integrating dietary metabarcoding data and assessing the spatial overlap between predicted suitable habitats and major agricultural regions.

Looking forward, we advocate for a transition from static, presence-based approaches to function-based, climate-informed conservation strategies. This includes identifying and protecting functional refugia, enhancing landscape connectivity to support range shifts, and embedding service-providing species into agroecological frameworks [76]. For *M. fuliginosus*, maintaining pest control services will require coordinated action across biodiversity policy, agricultural management, and spatial planning [77]. More broadly, this work demonstrates the value of integrating dietary ecology with species distribution models to predict changes in service delivery under global climate change. Applying this framework to other trophic or mutualistic taxa can help identify ecosystems at risk of functional erosion and guide the design of multifunctional, resilient landscapes [78]. As climate and land-use pressures intensify, conservation science must increasingly center on sustaining not only biodiversity but also the vital services it provides to human and natural systems [79].

## 5. Conclusions 

Our integrative analysis revealed that *M. fuliginosus*, a widespread insectivorous bat species in East Asia, maintains a functionally specialized diet strongly biased toward Lepidopteran pest species across distinct biogeographic regions. Despite this trophic specialization, climate projections indicate a substantial reorganization of its suitable habitat, characterized by poleward shifts and divergent patterns of landscape fragmentation under alternative emission scenarios. Notably, future low-emission trajectories (SSP1–2.6) support the retention and consolidation of climatically suitable core areas, whereas high-emission trajectories (SSP5–8.5) promote spatial decoupling through fragmentation and the loss of structural connectivity.

These findings highlight the dual importance of climatic stability and habitat configuration in sustaining the pest control services provided by bats. The observed alignment between trophic specialization and climatic refugia under low-emission scenarios suggests that climate mitigation could enhance the spatial resilience of bat-mediated ecosystem functions. Conversely, under more severe warming, the erosion of habitat cohesion may jeopardize functional continuity despite apparent range expansion.

From a conservation planning perspective, efforts to sustain bat-based pest control in agricultural landscapes should prioritize the preservation of stable core habitats and maintain connectivity among fragmented patches. This includes protecting forest–agriculture mosaics within projected refugial zones and integrating climate-informed spatial planning into ecosystem service management. Moreover, our findings underscore the value of linking functional traits with spatial modeling to anticipate where and how ecological functions may persist or erode in a changing world.

## Data Availability

Data will be made available on request.

## Supplementary Materials

The following supporting information can be downloaded at https://www.mdpi.com/article/10.3390/biology14111528/s1, Table S1. Geographic coordinates and metadata for occurrence records of *Miniopterus fuliginosus* used in SDMs. Table S2. Environmental variables used in species distribution models (SDMs). Table S3. Projected area change of suitable habitat under different climate scenarios. Table S4. Latitudinal and longitudinal shifts in habitat suitability centroids across scenarios. Table S5. Changes in landscape connectivity metrics under future climate scenarios. Figure S1. Trends in landscape connectivity metrics for *Miniopterus fuliginosus* under future climate scenarios. The panels show projected changes in (A) patch number, (B) area of the largest patch, (C) aggregation index, and (D) mean patch area between current conditions and future conditions for 2070 under the SSP1–2.6 and SSP5–8.5 scenarios. The results are shown across high, medium, and low suitability categories, indicating contrasting trends in fragmentation and spatial cohesion under alternative emission trajectories.
