# Supplementary material for: Climate-Driven Shifts in Bat Distributions Reveal Functional Reorganization and Spatial Mismatch Across Agroecosystems
Source: Biology (Basel). 2025 Oct 30;14(11):1528. doi: 10.3390/biology14111528 (PMC12650621; doi:10.3390/biology14111528)
Supplement: Supplementary file 1 [file biology-14-01528-s001.zip › Supplementary_FiguresS1.pdf]

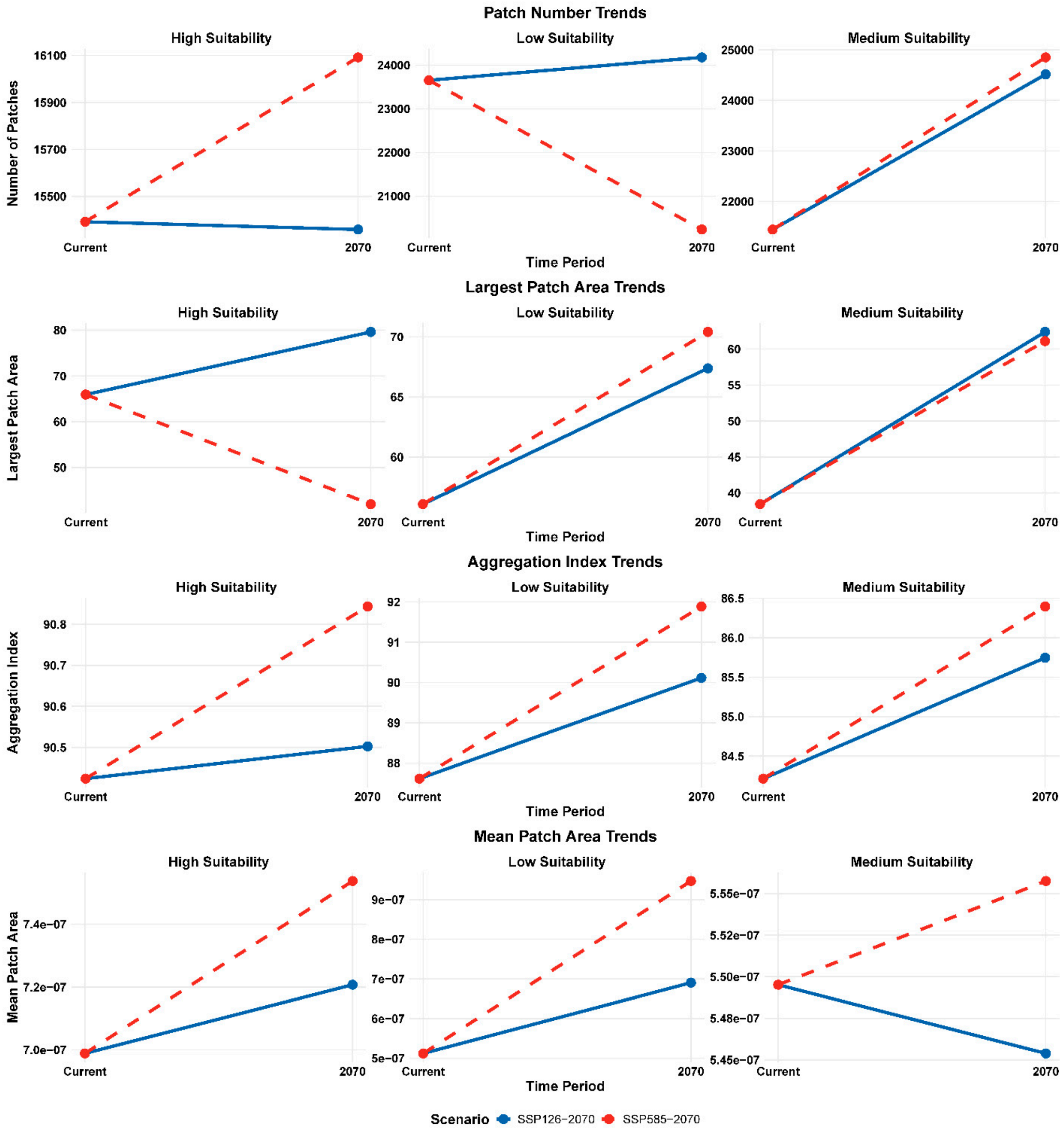

Figure S1. Trends in landscape connectivity metrics for *Miniopterus fuliginosus* under future climate scenarios. The panels show projected changes in **(A)** patch number, **(B)** area of the largest patch, **(C)** aggregation index, and **(D)** mean patch area between current conditions and future conditions for 2070 under the SSP1–2.6 and SSP5–8.5 scenarios. The results are shown across high, medium, and low suitability categories, indicating contrasting trends in fragmentation and spatial cohesion under alternative emission trajectories.
